# Cariprazine as a Dual-Action Agent in Substance-Related Psychosis: Clinical Remission and Functional Recovery

**DOI:** 10.1192/j.eurpsy.2026.10800

**Published:** 2026-07-31

**Authors:** D. Vlahović, S. Nadalin, D. Duraković

**Affiliations:** 1 Department of Psychiatry, University Hospital Center Sestre milosrdnice; 2 School of Medicine, Catholic University of Croatia, Zagreb; 3 General Hospital “Dr. Josip Benčević”, Slavonski Brod, Croatia

## Abstract

**Introduction:**

Cocaine-induced psychosis (CIP) can closely mimic primary psychotic disorders, complicating accurate diagnosis and treatment. Misdiagnosis may result in unnecessary long-term antipsychotic use and suboptimal outcomes. Cariprazine, a dopamine D3-preferring partial agonist, has shown promise in treating dual diagnosis patients due to its unique receptor profile.

**Objectives:**

To present a diagnostically challenging case of CIP initially misdiagnosed as schizophrenia and successfully treated with cariprazine monotherapy, leading to full symptomatic and functional recovery.

**Methods:**

A 27-year-old male presented with acute psychosis after a cocaine binge. He was diagnosed with first-episode schizophrenia and initiated on cariprazine 1.5 mg/day, titrated to 3 mg/day. Standard assessments included the Brief Psychiatric Rating Scale (BPRS), Cocaine Craving Scale (CCS), and Clinical Global Impression–Severity (CGI-S). He also received psychosocial interventions. A longitudinal reassessment was performed over a 12-month follow-up.

**Results:**

The patient showed rapid symptomatic improvement, with marked reduction in hallucinations, delusions, and craving by Day 7. He tolerated cariprazine well with no significant side effects. Over 12 months, he remained abstinent, free of psychotic symptoms, and achieved full functional recovery. Clinical reassessment led to a revised diagnosis of stimulant-induced psychosis.

**Conclusions:**

This case highlights the importance of diagnostic vigilance in acute psychosis with recent substance use. Cariprazine demonstrated efficacy and tolerability in a dual-diagnosis context, contributing to sustained remission and abstinence. Its pharmacological profile may offer benefits in managing CIP and warrants further investigation in controlled studies. Importantly, its D3 receptor partial agonism may have contributed to the reduction of cocaine craving and relapse risk, supporting its potential utility in targeting both psychotic symptoms and the underlying reward dysregulation in stimulant use disorders.

**Disclosure of Interest:**

None Declared

